# Supplementary material for: RrGT2, A Key Gene Associated with Anthocyanin Biosynthesis in Rosa rugosa, Was Identified Via Virus-Induced Gene Silencing and Overexpression
Source: Int J Mol Sci. 2018 Dec 14;19(12):4057. doi: 10.3390/ijms19124057 (PMC6321322; doi:10.3390/ijms19124057)
Supplement: Supplementary file 1 [file ijms-19-04057-s001.pdf]

# *RrGT2*, A Key Gene Associated with Anthocyanin Biosynthesis in *Rosa Rugosa*, Was Identified Via Virus-Induced Gene Silencing and Overexpression

Xiaoming Sui, Mingyuan Zhao, Zongda Xu, Lanyong Zhao and Xu Han

**Table S1.** Primers used in the present study.

| Primer Name | Sequence(5′-3′) | Description |
|---|---|---|
| 3′*RrGT2*-F-outer | GCAGAGAGGAACTAGAAGAGCTTGGG | |
| 3′*RrGT2*-F-inner | GCCAAGTTGATAGAGGACATGTGG | |
| B26 | GACTCGAGTCGACATCGATTTTTTTTTTTTTTTTT | 3′RACE for *RrGT2* |
| *RrGT2*-F | ATGACGCAACACCGCTTC | |
| *RrGT2*-R | CTAGGCTGGCAAACAGCCA | Full-length cDNA for *RrGT2* |
| p-*RrGT2*-F | ACTAGTATGACGCAACACCGCTTC | |
| p-*RrGT2*-R | GGTGACCCTAGGCTGGCAAACAGCCA | Generation for *pCAMBIA1304-RrGT2* |
| CpR-F | ATGGTAGATCTGACTAGTATGACGCAA | |
| CpR-R | TTCGAGCTGGTCACCCTAGG | Confirmation for *pCAMBIA1304-RrGT2* |
| TGR-F | GCTCTAGAATCAATCCTTCCCTGCAACTTG | |
| TGR-R | CCCTCGAGGATCTTTGTAGCCGTTGAAGTAG | Generation for *pTRV2-GFP- RrGT2* |
| *TRV1*-F | TTACAGGTTATTTGGGCTAG | |
| *TRV1*-R | CCGGGTTCAATTCCTTATC | RT-PCR for *TRV1* |
| *TRV2*-F | TGGGAGATGATACGCTGTT | Confirmation for recombinant silencing vector |
| *TRV2*-R | CCTAAAACTTCAGACACG | and RT-PCR for *TRV2* |
| *GFP*-F | ATGGTGAGCAAGGGCGAGGA | |
| *GFP*-R | CTTGTACAGCTCGTCCATGCC | RT-PCR for *GFP* |
| *RrGAPDH*-F | TTCTGCCTGCTCTCAATG | |
| *RrGAPDH*-R | TGCCTTCTTCTCAAGTCTG | qRT-PCR for *RrGAPDH* |
| q*RrGT2*-F | GTTGTTGGATTCCGGACGTC | |
| q*RrGT2*-R | CAAGCTCTTCTAGTTCCTCTCTGC | qRT-PCR for *RrGT2* |
| q*RrCHS*-F | ATGGTGACCGTCGAGGAAGTC | |
| q*RrCHS*-R | GCCTTGTGCTCGCTCTTAGTG | qRT-PCR for *RrCHS* |
| q*RrCHI*-F | ATGGCTCAATCAGTTACCGGA | |
| q*RrCHI*-R | GCACGGCCTTATCCTCCAAGT | qRT-PCR for *RrCHI* |
| q*RrF3H*-F | GAGATGTGACCAAGCAGTACA | |
| q*RrF3H*-R | TCAACGCCTCCTTCTCTAAAC | qRT-PCR for *RrF3H* |
| q*RrF3′H*-F | CTTGGTCATGGCTATTCCTTC | |
| q*RrF3′H*-R | CCATTGATGAGGTTGAGGTT | qRT-PCR for *RrF3′H* |
| q*RrDFR*-F | GTGAAGATGACTGGTTGGATG | |
| q*RrDFR*-R | CGAGAGTTGGGATAATGGTGA | qRT-PCR for *RrDFR* |
| q*RrANS*-F | CCAACTCCATCGTCATGCAC | |
| q*RrANS*-R | CAGCGGCTTGAGGATGATCTT | qRT-PCR for *RrANS* |
| *NtACT*-F | AGGGTTTGCTGGAGATGATG | |
| *NtACT*-R | CGGGTTAAGAGGTGCTTCAG | qRT-PCR for *NtACT* |

* Restriction enzyme site are underlined.

**Table S2.** Anthocyanin contents in the flowers of *R. rugosa* 'Zizhi' and *R. davurica* subjected to different VIGS treatments ($\mu g \cdot g^{-1}$ FW).

| Name | treatments | Cy3G | Cy3G5G | Pn3G | Pn3G5G | Pg3G | Pg3G5G | Dp3G | Dp3G5G |
|---|---|---|---|---|---|---|---|---|---|
| *R. rugosa* 'Zizhi' | Control | 13.05 ± 0.04A | 298.38 ± 3.85A | 17.63 ± 0.12A | 1773.93 ± 4.31A | 5.04 ± 0.09A | 52.88 ± 0.45A | - | - |
| | TRV-GFP | 11.86 ± 0.70A | 301.14 ± 1.57A | 17.33 ± 0.07A | 1660.26 ± 13.07B | 4.82 ± 0.13A | 45.32 ± 1.23B | - | - |
| | TRV-GFP-*RrGT2* | 3.29 ± 0.22B | 59.92 ± 0.28B | 6.71± 0.20B | 189.41 ± 0.54C | - | 2.28 ± 0.23C | - | - |
| *R. davurica* | Control | 56.72 ± 0.20A | 2590.18 ± 2.45A | 2.55 ± 0.03A | 40.28 ± 0.09A | 7.20 ± 0.01A | 25.7 ± 0.36B | - | - |
| | TRV-GFP | 55.59 ± 0.26B | 2565.85 ± 3.26B | 2.50 ± 0.01A | 40.36 ± 0.11A | 7.19 ± 0.01A | 28.13 ± 0.27A | - | - |
| | TRV-GFP-*RrGT2* | 16.28 ± 0.13C | 425.77 ± 3.86C | - | 1.79 ± 0.03B | 0.68 ± 0.02B | 7.23 ± 0.04C | - | - |

* Data are the mean values ± SE of three independent replicates.Different upper case letters represent significant difference which is calculated using LSD analysis at the level of $P < 0.01$. '-' means that no corresponding anthocyanin was detected.

**Table S3.** Anthocyanin contents in the flowers of transgenic tobacco ($\mu g \cdot g^{-1}$ FW).

| Name | treatments | Cy3G | Cy3G5G | Pn3G | Pn3G5G | Pg3G | Pg3G5G | Dp3G | Dp3G5G |
|---|---|---|---|---|---|---|---|---|---|
| OE-*RrGT2* in Tobacco | Control | 28.6367 ± 0.024D | - | - | - | - | - | - | - |
| | P | 28.3533 ± 0.029D | - | - | - | - | - | - | - |
| | T1 | 54.47 ± 0.036B | - | - | - | - | - | - | - |
| | T3 | 67.7667 ± 0.038A | - | - | - | - | - | - | - |
| | T6 | 51.6667 ± 0.353C | - | - | - | - | - | - | - |

* Data are the mean values ± SE of three independent replicates.Different upper case letters represent significant difference which is calculated using LSD analysis at the level of $P < 0.01$. '-' means that no corresponding anthocyanin was detected.

**Table S4.** Anthocyanin contents in the flowers of transgenic tobacco subjected to different VIGS treatments ($\mu g \cdot g^{-1}$ FW).

| Name | treatments | Cy3G | Cy3G5G | Pn3G | Pn3G5G | Pg3G | Pg3G5G | Dp3G | Dp3G5G |
|---|---|---|---|---|---|---|---|---|---|
| VIGS of OE-*RrGT2* Tobacco Plants | Control | 67.7667 ± 0.038A | - | - | - | - | - | - | - |
| | TRV-GFP | 65.2267 ± 0.89B | - | - | - | - | - | - | - |
| | V1 | 11.71 ± 0.032D | - | - | - | - | - | - | - |
| | V2 | 15.3533 ± 0.029C | - | - | - | - | - | - | - |
| | V3 | 7.77 ± 0.064E | - | - | - | - | - | - | - |

* Data are the mean values ± SE of three independent replicates.Different upper case letters represent significant difference which is calculated using LSD analysis at the level of P < 0.01. '-' means that no corresponding anthocyanin was detected.

**Table S5.** Silencing efficiency of VIGS in *R. rugosa* 'Zizhi' and *R. davurica.*

| Name | | TRV-GFP | TRV-GFP-*RrGT2* |
|---|---|---|---|
| *R. rugosa* 'Zizhi' | Total | 20 | 20 |
| | GFP positive | 15 | 13 |
| | *RrGT2* silencing | - | 13 |
| | GSEI | - | 65% |
| *R. davurica* | Total | 20 | 20 |
| | GFP positive | 19 | 17 |
| | *RrGT2* silencing | - | 17 |
| | GSEI | - | 85% |

* GSEI, gene silencing efficiency of all infected plants.

*2.S1 Construction and Verification of Recombinant Virus Vector pTRV2-GFP-RrGT2*

The virus vector pTRV2-GFP and the specific fragment of the *RrGT2* gene were respectively digested (Figure S1B) and then were combined together , according to the preset viral vector recombination technology route (Figure S1A). Then the PCR (Figure S1C)was verified by specific primers (Table S1), and the base mutation and deletion were verified by double enzyme digestion (Figure S1D)and sequencing. All of the results above indicated that the recombinant virus vector pTRV2-GFP-*RrGT2* was successfully constructed.

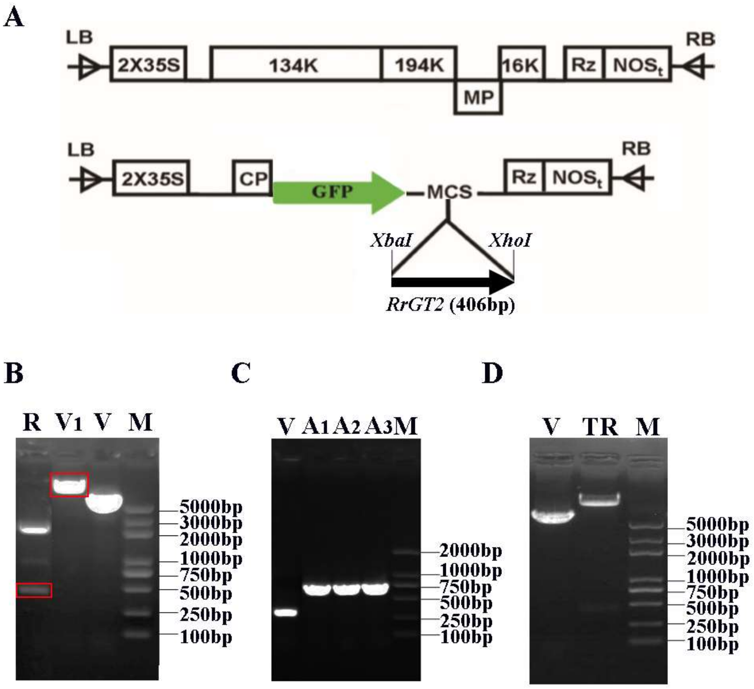


**Figure S1.** Construction and validation of the recombinant virus vector TRV-GFP-*RrGT2*. **(A)** Schematic diagram of the construction of TRV-GFP-*RrGT2*. **(B)** The virus vector and target gene fragment were digested separately. M: Marker; V: The plasmid of the empty virus vector was used as the control; V1: The empty virus vector was digested by double enzymes; R: The plasmid carrying the target gene fragment was digested by two enzymes. The red box indicates the portion that needs to be retained for subsequent operations. **(C)** The result of PCR verification. Three repeated PCR assays were performed on the recombinant virus vector and the results showed positive. M: Marker; V: The empty virus vector was used as the control; A1,A2,A3 was the three repetitions which were larger than the empty virus vector due to the

inserted *RrGT2* fragment. **(D)** Double enzyme digestion was used to verify the plasmid of the recombinant vector. M: Marker; V: The plasmid of the empty virus vector was used as the control; TR: The plasmid of the recombinant vector was digested into two correct parts.

*2.S2 Construction and Verification of Recombinant Expression Vector pCAMBIA1304-RrGT2*

Similar to the construction process of the recombinant virus vector , the empty vector and target gene were digested separately (Figure S2B) and linked together according to the pre-designed vector recombination technique route (Figure S2A). In order to verify whether the recombination was successful, we designed a pair of specific primers (Table S1) to detect the transformation results by PCR, and the results showed positive (Figure S2C). In order to eliminate the possibility of false positivity and to further verify whether the target gene had been mutated or deleted during the recombination, we identified the target gene by double enzyme digestion and sequencing. The results of double enzyme digestion (Figure S2D) showed that the recombinant vector was cut into two distinct fragments: one part of the original vector and the other part of the target gene. The plasmid of the empty vector was used as the control. The sequencing results also showed that there was no base mutation and deletion in the target gene fragment of the embedded vector. The results above showed that the recombinant expression vector *pCAMBIA1304-RrGT2* was constructed successfully.

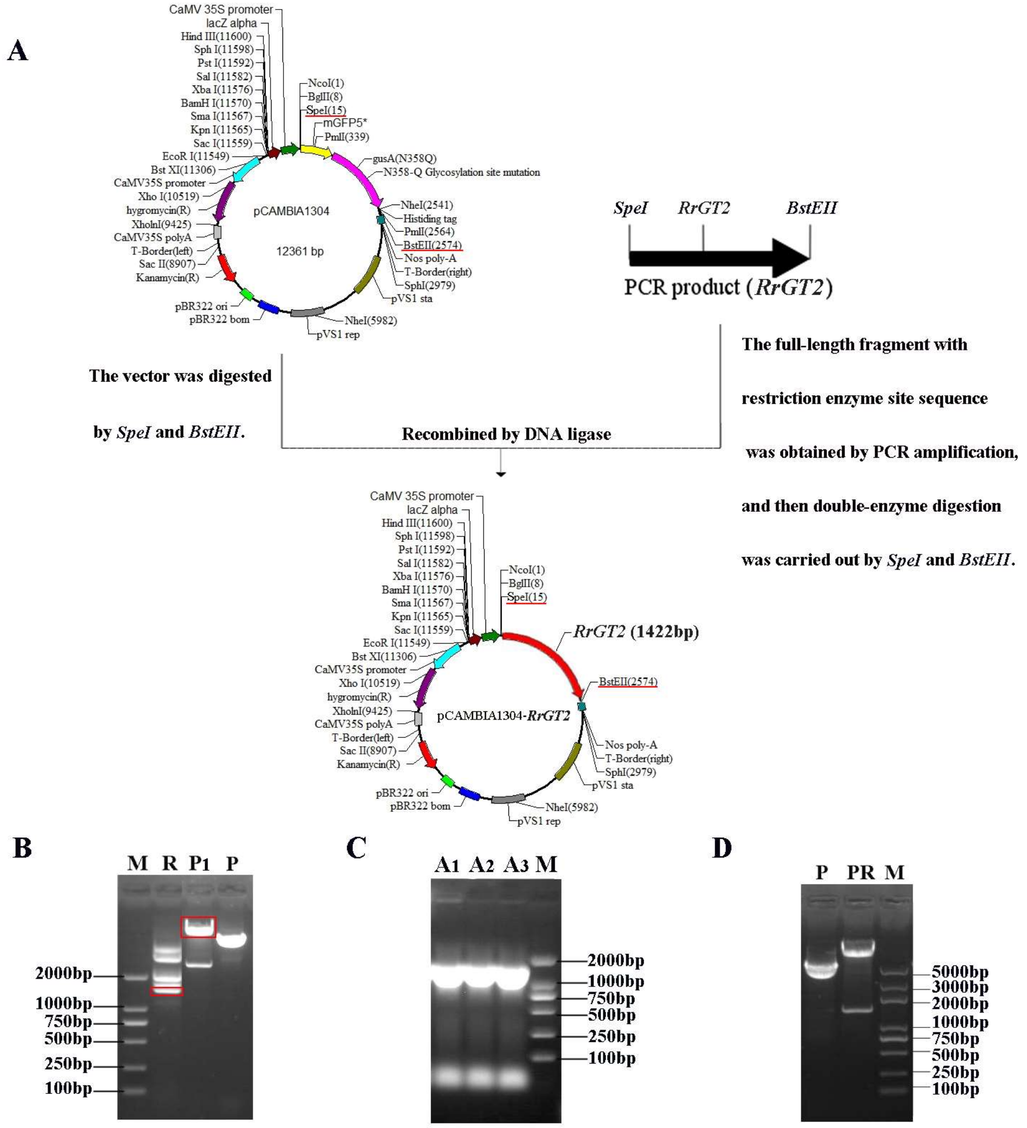


**Figure S2.** Construction and validation of the recombinant expression vector pCAMBIA1304-*RrGT2*. **(A)** Schematic diagram of the construction of pCAMBIA1304-*RrGT2*. **(B)** The vector and target gene were digested separately. M: Marker; P: The plasmid of the empty vector pCAMBIA1304 was used as the control; P1: The empty vector was digested by double enzymes; R: The plasmid carrying the target gene was digested by two enzymes. The red box indicates the portion that needs to be retained for subsequent operations. **(C)** The result of PCR verification. Three repeated PCR assays were performed on the recombinant vector and the results showed positive. M: Marker; A1,A2,A3 was the three repetitions. **(D)** Double enzyme digestion was used to verify the plasmid of the recombinant vector. M: Marker; P: The plasmid of the empty vector pCAMBIA1304 was used as the control; PR: The plasmid of the recombinant vector was digested into two correct parts.
